# Load-velocity relationships and predicted maximal strength: A systematic review of the validity and reliability of current methods

**DOI:** 10.1371/journal.pone.0267937

**Published:** 2022-10-27

**Authors:** Kieran J. Marston, Mitchell R. L. Forrest, Shaun Y. M. Teo, Sean K. Mansfield, Jeremiah J. Peiffer, Brendan R. Scott

**Affiliations:** 1 Centre for Healthy Ageing, Murdoch University, Murdoch, Western Australia, Australia; 2 Murdoch Applied Sports Science Laboratory, Murdoch University, Murdoch, Western Australia, Australia; 3 Telethon Kids Institute, The University of Western Australia, Perth, Australia; University of Rome, ITALY

## Abstract

Maximal strength can be predicted from the load-velocity relationship (LVR), although it is important to understand methodological approaches which ensure the validity and reliability of these strength predictions. The aim of this systematic review was to determine factors which influence the validity of maximal strength predictions from the LVR, and secondarily to highlight the effects of these factors on the reliability of predictions. A search strategy was developed and implemented in PubMed, Scopus, Web of Science and CINAHL databases. Rayyan software was used to screen titles, abstracts, and full texts to determine their inclusion/eligibility. Eligible studies compared direct assessments of one-repetition maximum (1RM) with predictions performed using the LVR and reported prediction validity. Validity was extracted and represented graphically via effect size forest plots. Twenty-five eligible studies were included and comprised of a total of 842 participants, three different 1RM prediction methods, 16 different exercises, and 12 different velocity monitoring devices. Four primary factors appear relevant to the efficacy of predicting 1RM: the number of loads used, the exercise examined, the velocity metric used, and the velocity monitoring device. Additionally, the specific loads, provision of velocity feedback, use of lifting straps and regression model used may require further consideration.

## Introduction

Of the acute resistance training variables which can be manipulated (e.g., time under tension, inter-set/inter-repetition recovery duration, range of motion [1]), the load lifted is amongst the most important [2]. Typically, the loads lifted are prescribed relative to the pre-determined maximal load an individual can lift for 1-repetition (1-repetition maximum; 1RM) [2,3]. For example, loads of ≥85% 1RM are often recommended in order to optimise muscular strength [3]. Direct 1RM assessment is a valid and reliable method to determine maximal strength [4], but can be time-consuming and is physically demanding [5]. This may be particularly true when determining 1RM for multiple exercises or large groups of athletes [6], which could render regular direct 1RM assessments as unviable in some situations.

Recently, with the increased prevalence of velocity-monitoring technology in strength and conditioning practice [7,8], research has begun to examine the merit of using the load-velocity relationship (LVR) to predict 1RM [6,9,10]. The development of individualised LVRs involves plotting the inverse-linear relationship between a series of incrementally heavy loads and the velocity at which each can be lifted [11]. While the number of different loads used and what specific loads are lifted differs between studies, all methods of using LVRs to predict 1RM are submaximal. Therefore, these predictions may allow for more frequent assessment of an individuals’ maximal strength, while moderating the physical demands to the participant.

Currently, there are three methods of predicting 1RM using LVRs: the minimal velocity threshold (MVT), load at zero velocity (LD0), and force-velocity method (FV). The MVT method is based on the notion that the velocity of 1RM, commonly referred to as the MVT, is consistent (i.e., for the same individual and exercise). Subsequently, the linear regression equation for an individual’s LVR can be solved for the MVT to predict 1RM [6]. The LD0 method involves solving the regression of the LVR for the load corresponding with a velocity of 0 m·s^-1^ [12]. The final method proposed in the literature is the FV method. This method involves monitoring repetition force in addition to velocity and subsequently determining the interception of an individual’s FV and weight-velocity relationship (calculated by multiplying the loads used by 9.81m·s^-1^) [13]. Three main velocity metrics are typically used to develop LVRs; mean concentric velocity (MCV), peak concentric velocity (PCV) and mean propulsive velocity (MPV) [11,14]. The MCV and PCV refer to the average and maximal velocity achieved through the entire concentric phase, respectively [11,14]. Alternatively, MPV refers to the average velocity of the accelerative component of the concentric phase (i.e., the start of the concentric phase until the acceleration is < −9.81m·s^-1^) [11,14]. Early research theorized that while PCV may be a relevant measure for ballistic exercises such as jumps and throws, MCV is likely superior for non-ballistic exercises as it is representative of the entire concentric phase [14,15]. In these non-ballistic exercises however, individuals typically decelerate towards the end of the concentric phase to maintain balance [16]. Thus, researchers have suggested that the MPV, which disregards this “breaking phase” of the lift, may improve the reliability of LVRs developed using this metric [16].

While the validity of each of these methods has been discussed favourably in at least one study, conflicting outcomes are common. Within individual studies, these inconsistencies are most often attributed to the population tested [15,17], exercise being assessed [17], equipment being used [6], loads lifted [18], the device used to quantify repetition velocity [19] and the specific velocity metric examined [14,15]. To address these inconsistencies in the current literature, it is appropriate that a systematic review is completed to guide future endeavours of practitioners and researchers alike. Therefore, the aim of this systematic review is to provide a comprehensive assessment of factors that contribute to the validity of using LVRs to predict 1RM, and to present specific contexts which will enhance the validity of these 1RM predictions. Secondarily, we will report on the reliability of LVR-derived 1RM predictions. We hypothesise that the validity of 1RM predictions will be influenced by parameters such as the number of loads used for the prediction model, the exercise examined, the velocity metric used, and the velocity monitoring device used.

## Methods

### Eligibility criteria

We included full-text, peer-reviewed, original research investigating the validity of predicting 1RM from LVRs using MVT, LD0 and/or FV methods. Eligible studies compared direct assessments of 1RM with 1RM predictions performed using the LVR and reported the validity of 1RM predictions. We excluded unpublished studies, editorials, books, letters, conference proceedings and reviews. Studies in older (i.e., aged ≥ 65 years) or clinical population were not considered for inclusion. Since maximal strength is markedly reduced in fatigued individuals (i.e., impacting on 1RM assessment validity) [20], studies that investigated LVR-based 1RM predictions in individuals experiencing neuromuscular fatigue were also excluded. Further, we excluded studies examining predictions of power/ballistic exercises (e.g., jump squat, weightlifting movements). All included articles were written in the English language. There were no restrictions on country of origin, sex of participants, or the training experience of participants. In accordance with the previous research, measured 1RM was defined as the maximal load that an individual can lift for 1-repetition with safe technique [4]. All available methods of predicting 1RM using the relationship between the loads used and the velocity at which it can be lifted were considered for this review.

### Information sources

A comprehensive search strategy was developed and implemented in the following databases from inception (June 15, 2020) to August 21, 2020: PubMed, Scopus, Web of Science and CINAHL. The search results were updated August 18, 2021. The search syntax was initially developed for PubMed (Table 1) and then adapted for use in the other databases. This syntax included terms relevant to LVRs and maximal strength. The reference lists of all included articles were manually searched to identify any remaining studies. All search results were extracted and imported into a reference manager (EndNote X9, Thomson Reuters, Philadelphia, PA, USA). Approval from the institutional ethics committee was not required for the completion of this review.

**Table 1:** Search terms used for literature search.

Search string 1	Search string 2
(“Load-velocit*” OR “load velocit*” OR “force-velocit*” OR “force velocit*”)	(“1RM” OR “1-RM” OR “1 RM” OR “1-rep* max*” OR “1 rep* max*” OR “one-rep* max*” OR “one rep* max*” OR “Strength”)

*Note*: Search strings 1 and 2 were combined with using Boolean operator AND to create one larger search string.

### Study selection

Using Rayyan software for systematic reviews [21], two review authors (KJM and MRLF) independently screened the titles and abstracts of 2308 identified articles to determine their eligibility. The full texts of 60 eligible studies were then evaluated independently by the two review authors (KJM and SYMT) with 25 studies included in the qualitative synthesis. Any conflicts regarding article inclusion at this stage were resolved through discussion with a third review author.

### Data collection process

Two review authors (KJM and SYMT) used a customized data extraction form to collect the following information from each study: participant sex, age, height, body mass, resistance training experience, maximal strength levels, the 1RM prediction method used, the velocity and/or force measurement device, the specific velocity variable used, the number of points and which loads were used to develop the LVR and study results for validity outcomes. If available, reliability outcomes were also extracted. Disagreements between the two review authors (KJM and SYMT) regarding any of the aforementioned information were resolved through discussion with a third review author. Effect sizes (Cohen’s d and Hedge’s g, [ES]) were calculated from the reported means if not provided in-text. For graphical representation, effect sizes were normalised to positive values to interpret the overall validity of LVR predictions (i.e., rather than evaluating the overestimation or underestimation of each model). Previous recommendations for acceptable reliability (i.e., ES < 0.30) were included to interpret the reliability of the included studies [18,19]. Although the context-specific interpretation of reliability (i.e., intraclass correlation coefficient [ICC]) has not been examined extensively, reliability via ICC was interpreted as ‘excellent’ (≥ 0.90), ‘good’ (0.75 to 0.90, ‘moderate’ (0.50 to 0.75) or ‘poor’ (≤ 0.50) [22].

### Assessment of reporting quality

The reporting quality of the research included in this review was assessed independently by two review authors (MRLF and SKM) using a modified version of the Downs and Black checklist (Table 2) [23]. This method is valid for assessing the methodological reporting quality for observational study designs and is commonly used in systematic reviews pertaining to sports science research [24–26]. The study quality was assessed against nine items and assigned a score of ‘0’ if the criteria was not met or could not be determined by the reviewing authors and a score of ‘1’ if the criteria for the item had been met. Any disagreements between the two reviewing authors on reporting quality were resolved through discussion with a third review author (KJM). With no reference ranges available for modified checklists, previously published ranges were used to develop appropriate relative scoring [27]. As such, scores were interpreted as ‘excellent’(8 to 9), ‘good’ (6 to 7), ‘fair’ (5 to 6) or ‘poor’ (≤4). No studies were excluded due to ‘poor’ reporting quality.

**Table 2:** Assessment of reporting quality of included studies.

Study (year)	Downs and Black Checklist items									
	Reporting						Internal Validity			
	1	2	3	6	7	10	16	18	20	Σ
Balsalobre-Fernández et al.(2018) [28]	1	1	1	1	0	1	1	1	1	8
Balsalobre-Fernández et al.(2019) [29]	1	1	0	1	1	1	1	0	1	7
Balsalobre-Fernández et al.(2021) [37]	1	1	1	1	0	1	1	0	1	7
Banyard et al.(2017) [6]	1	1	1	1	1	1	1	0	1	8
Benavides-Ubric et al. (2020) [38]	1	1	1	1	0	0	1	1	1	7
Caven et al. (2020) [43]	1	1	1	1	1	1	1	0	1	8
Fernandes et al. (2021) [39]	1	1	0	1	0	1	1	0	1	6
Garcia-Ramos et al.(2018a) [9]	1	1	1	1	0	0	1	1	1	7
Garcia-Ramos et al.(2019) [30]	1	1	1	1	1	1	1	1	1	9
Hughes et al.(2019) [36]	1	1	1	1	0	0	1	1	1	7
Janicijevic et al. (2021) [40]	1	1	1	1	0	1	1	0	1	7
Jidovtseff et al.(2011) [21]	1	1	0	1	0	0	1	0	1	5
Jiménez-Alonso et al.(2020) [31]	1	1	1	1	1	1	1	1	1	9
Jukic et al.(2020) [18]	1	1	1	1	0	1	1	1	1	8
Lake et al.(2017) [32]	1	1	1	1	1	0	1	1	1	8
Loturco et al.(2016) [35]	1	1	0	1	1	0	1	0	1	6
Loturco et al.(2017) [17]	1	1	0	1	1	0	1	0	1	6
Loturco et al.(2018) [15]	1	1	0	1	0	0	1	0	1	5
Pérez-Castilla et al.(2019a) [42]	1	1	0	1	1	0	1	1	1	7
Pérez-Castilla et al.(2019b) [19]	1	1	0	1	0	1	1	0	1	6
Pérez-Castilla et al.(2020) [33]	1	1	0	1	1	1	1	1	1	8
Picerno et al.(2016) [13]	1	1	1	1	1	1	1	1	1	9
Ruf et al.(2018) [34]	1	1	1	1	0	0	1	0	1	6
Sayers et al.(2018) [44]	1	1	0	1	1	0	1	1	1	7
Thompson et al. (2021) [41]	1	1	1	1	1	1	1	1	1	9

*Note*: Σ = sum.

## Results

### Study selection

**Fig 1:**
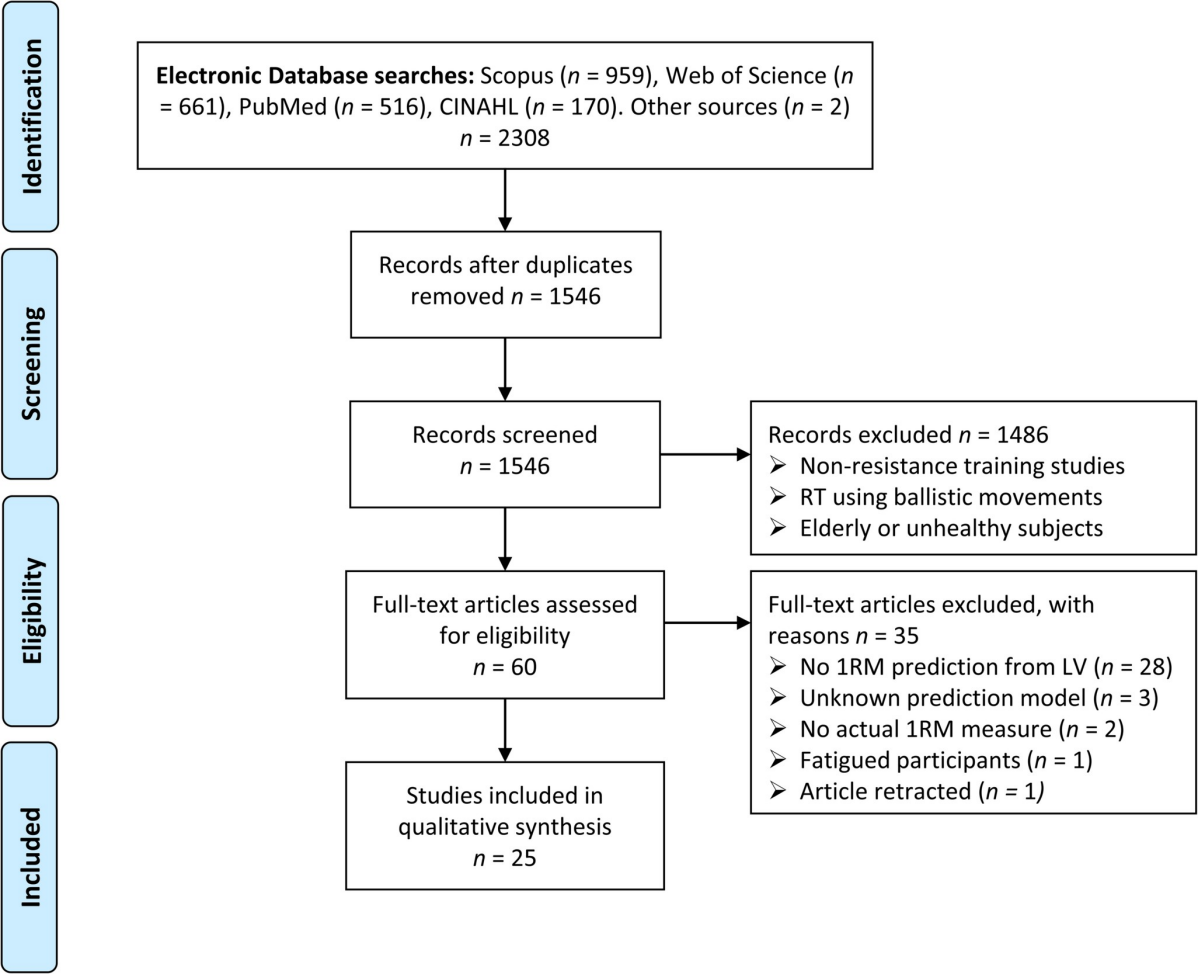
Flow diagram of study selection process.

The study selection procedure has been outlined in a flow diagram presented in Fig 1. The initial search strategy yielded 2308 articles. After removing duplicates, 1546 articles were included in the title/abstract screening, and 60 studies were retained for full-text review.

### Research reporting quality

The methodological reporting quality of the research examining the validity and/or reliability of predicting 1RM from LVRs was relatively high (mean ± standard deviation = 7.2 ± 1.2 [range = 5 to 9]) when appraised using the modified Downs and Black checklist [23]. Items that were not consistently achieved were questions 3 (adequately defined population, n = 10), 7 (reporting on distribution of data, n = 12), 10 (reporting exact *p* values, n = 11) and 18 (was the statistical analysis appropriate and rigorous, n = 12). Where *p* values were provided as a range, and the specific value corresponding to each comparison could not be determined, question 10 was scored a “0”. In the context of this review, sex, age, training experience and strength levels of the population were considered essential descriptors to score “1” for question 3. Data had to be examined on the individual level (i.e., limits of agreement and Bland-Altman method), rather than based on a comparison between the means, for the statistics used to be deemed appropriate. This decision was made given that a number of previous studies have highlighted no differences between mean predicted and measured 1RM, yet still substantial errors at the individual level.

### Study characteristics

The 25 eligible studies included in this systematic review comprised a total of 842 participants, three different 1RM prediction methods, 44 different loading models (i.e., how many loads used for prediction and at what percentage of 1RM each load was lifted), 16 different exercises, and 12 different velocity monitoring devices (Table 3).

**Table 3:** Descriptive statistics of included studies.

Study (year)	n	Sex; Age (mean±SD)	Height (mean±SD); body mass (mean±SD)	Training experience (mean±SD)	Exercise (modality)	Phase assessed	Maximal strength (mean±SD)
Balsalobre-Fernández et al.(2018) [28]	10	Male; 26.5±6.5 y	1.77±0.10 m; 86.0±24.3 kg	≥ 4 y	Bench press (FW)	ISO	111.7±39.7 kg
Balsalobre-Fernández et al.(2019) [29]	15	Male; 33.6±9.3 y	NS; NS	≥ 2 y	Leg extension (double and single legged [M])	ISO	NS
Balsalobre-Fernández et al.(2021) [37]	116	Male; 21.0±4.10 y	1.76±0.06 m; 73.3±14.0 kg	≥ 1 y	Bench press (SM)	ISO/CO	T1 = 73.7±18.2 kg T2 = 70.3±22.9 kg
Banyard et al.(2017) [6]	17	Male; 25.4±3.3 y	1.81±0.06 m; 81.8±9.9 kg	5.9±2.9 y	Back squat (FW)	ISO	140.3±27.2 kg
Benavides-Ubric et al. (2020) [38]	50	Male; 23.8±3.6 y	1.78±0.06 m; 78.2±8.3 kg	≥ 2 y	Deadlift (FW)	CO	T1 = 139.3±16.4 kg T2 = 140.0±16.0 kg
Caven et al. (2020) [43]	17	Female; 17.8±1.3 y	NS; 69.1±9.6 kg	≥ 1 y	Bench press (FW) Squat (FW)	ISO	BP = 38.6±7.5 kg SQ = 86.5±14.7 kg
Fernandes et al. (2021) [39]	40	Male; YG (20) = 21.0±1.6 y MG (20) = 42.6±6.7 y	YG = NS; 85.9±12.8 kg MG = NS; 82.3±11.2 kg	YG = 4.5±1.1 y MG = 16.9±11.4 y	Bench press (SM) Bent-over-row (SM)	ISO	NS NS
Garcia-Ramos et al.(2018a) [9]	30	Male; 21.2±3.8 y	1.78±0.07 m; 72.3±7.3kg	≥ 2 y	Bench press (SM)	ISO/CO	78.1±13.0 kg
Garcia-Ramos et al.(2019) [30]	26	Male; 20.5±2.9 y	1.76±0.07 m; 75.7±9.3 kg	6.1±3.9 y	Prone row (FW)	ISO	T1 = 89.8±13.4 kg T2 = 90.1±12.1 kg
Hughes et al.(2019) [36]	20	Male; 24.3±2.9 y	1.80±0.05 m; 84.2±10.5 kg	≥ 2 y	Back squat (FW)	ISO	151.1±25.7 kg
Janicijevic et al. (2021) [40]	86	Male; 20.9±4.2 y	1.73±0.03 m; 74.3±15.6 kg;	1.3±2.4 y	Bench press (SM)	ISO/CO	T1 = 61.6±17.5 kg T2 = 66.3±18.3 kg
Jidovtseff et al.(2011) [21]	112	Male (90), Female (22); 23.0±4.0 y	1.77±0.11; 72.0±14.0 kg	NS	Bench press (SM)	ISO	60.0±19.0 kg
Jiménez-Alonso et al.(2020) [31]	15	Male; 20.5±3.0 y	1.75±0.06 m; 74.3±8.8 kg	1.6±0.9 y	Bench press (FW)	ISO	80.2±9.5 kg
Jukic et al.(2020) [18]	18	Male; 24.4±2.3 y	1.82±0.05 m; 86.4±8.3 kg	≥ 1 y	Deadlift (FW; no straps [N] and with straps [W])	ISO	N; 162.0±26.9 kg W; 179.0±29.9 kg
Lake et al.(2017) [32]	12	Male; 20.3±0.6 y	1.80±0.08 m; 85.9±18.4 kg	≥ 1 y	Deadlift (FW)	ISO	182.1±21.2 kg
Loturco et al.(2016) [35]	64	Male; SJ = 23.0±4.1 y, AFR = 23.0±3.9 y, SA = 25.1±3.5 y, CA = 22.8±2.7 y	SJ = 1.80±0.05 m; 78.7±6.2 kg AFR = 1.83±0.06 m; 87.8±12.3 kg SA = 1.78±0.07 m; 74.4±4.6 kg CA = 1.81±0.05 m; 75.1±5.3 kg	NS	Half squat (SM)	ISO	SJ = 163.5±15 kg AFR = 167.8±23.7 kg SA = 122.3±12.1 kg CA = 124.3±8.5 kg
Loturco et al.(2017) [17]	36	Male; RU = 24.7±4.7 y, RS = 24.0±3.1 y, CA = 23.8±2.4 y	RU = 1.82±0.07 m; 97.4±10.2 kg RS = 1.81±0.07 m; 88.8±6.4 kg CA = 1.79±0.06 m; 74.1±4.3kg	NS	Bench press (FW) Bench press (SM)	ISO	109.7±21.4 kg 118.1±20.5 kg
Loturco et al.(2018) [15]	30	Male; RU = 20.5±4.6 y, CA = 30.3±9.9 y	RU = 1.75±0.22 m; 93.3±25.4 kg CA = 1.77±0.07 m; 88.3±18.7 kg	NS	Bent over row (FW) Bent over row (SM) Prone row (FW)	ISO/CO	84.3±13.0 kg 88.5±13.1kg 84.1±9.9 kg
Pérez-Castilla et al.(2019a) [42]	23	Male (12); 20.8±2.5 y. Female (11); 20.2±1.1 y	Male = 1.79±0.06 m; 78.9±10.7 kg Female = 172.2±0.05 m; 65.3±4.4 kg	NS	Lat pulldown (M) Seated cable row (M)	ISO	Male = 78.1±14.0 kg Female = 46.1±7.3 kg Male = 74.4±14.2 kg Female = 44.1±6.2 kg
Pérez-Castilla et al.(2019b) [19]	11	Male; 22.5±1.9 y	1.75±0.06 m; 75.2±7.2 kg	NS	Bench press (SM)	ISO	83.8±12.3 kg
Pérez-Castilla et al.(2020) [33]	20	Male; 22.5±3.7 y	1.78±0.06 m; 77.9±13.1 kg	NS	Bench press (SM)	ISO	81.0±3.0 kg
Picerno et al.(2016) [13]	37	Male (27), Female (10); 23.9±3.1 y	Male = 1.78±0.05 m; 75.6±7.5 kg Female = 1.65±0.06 m; 59.3±5.9 kg	Untrained	Chest press (M) Leg press (M)	CO	99.5±27.0 kg 249.3±60.2 kg
Ruf et al.(2018) [34]	11	Male; 23.6±1.4 y	1.80±0.06 m; 85.6±6.2 kg	3.2±0.9 y	Deadlift (FW)	ISO	T1 = 174.7±26.9 kg T2 = 176.0±25.7 kg
Sayers et al.(2018) [44]	12	NS; NS	NS; 80.8±5.7 KG	≥ 1 y	Bench press throw (SM)	ISO	84.0±18.0 kg
Thompson et al. (2021) [41]	14	Male; 26.0±3.8 y	1.75±0.05 m; 82.5±9.4 kg	≥ 1 y	Squat (FW)	ISO	157.0±19.4 kg

*Note*: **1RM =** one repetition maximum, **AFR =** American football and rugby athletes, **CA =** combat athletes, **CO =** concentric phase only, **FW =** free-weight, **ISO** = isotonic, **M =** machine, **MG =** middle-aged group, **N =** deadlift without straps, **RS =** rugby sevens athletes, **RU =** rugby union athletes, **SA =** soccer athletes, **SJ =** sprinting and jumping athletes, **SM =** Smith Machine, **T1 =** trial one, **T2 =** trial two, **W =** deadlift with straps, **YG =** young group.

**Fig 2:**
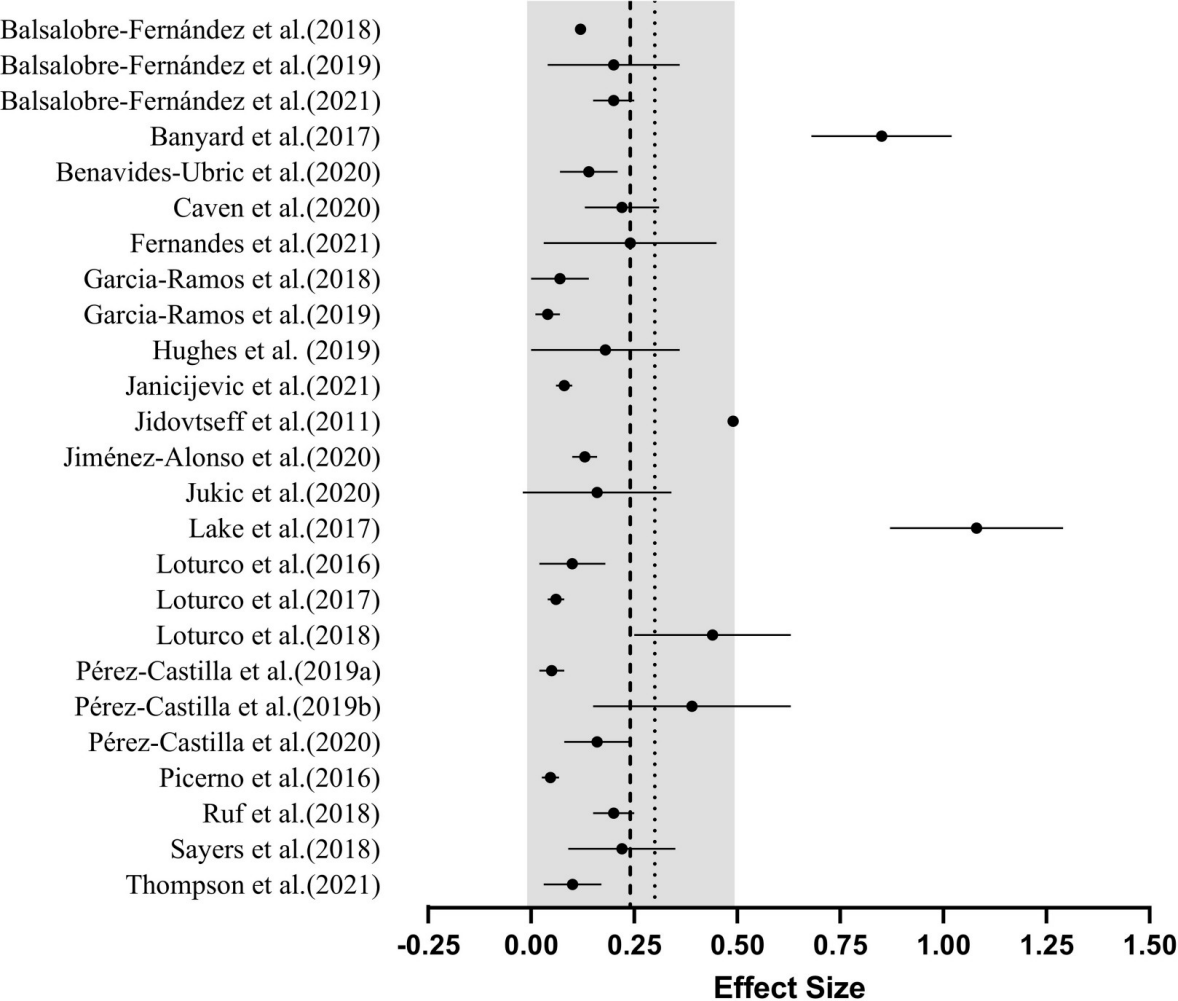
Overall mean (●) effect sizes (±SD), grouped average (---) and recommended maximum effect size (∙∙∙) lifor magnitude of difference between predicted and actual 1RM. The grey band represents the standard deviation of the pooled effect sizes. *Note*: Effect sizes closer to zero indicate greater validity (i.e., smaller difference between actual and predicted 1RM).

The grouped effect sizes for magnitude of difference between predicted and actual 1RM are presented graphically in Fig 2. Twenty studies (80%) included male participants only [6,9,15,17–19,28–41], three studies (12%) feature both male and female participants [12,13,42], one study (4%) included only females participants [43] and one study (4%) did not describe the sex of participants [44]. The training experience of participants also varied substantially across each of the studies included. Largely, the studies included in this review (36%) recruited participants who had been performing frequent resistance training for over two years [6,9,28–30,34,36,38,39]. Seven studies (28%) recruited participants with a minimum of one year of regular resistance training [18,31,32,37,39,43,44] and one study (4%) intentionally recruited untrained individuals [13]. Further, eight studies (37%) did not state a minimal level of prior training experience [12,15,17,19,33,35,40,42]. The MVT method (Table 4) was examined 22 times (88%) [6,9,15,17–19,28–43], the LD0 method (Table 5) three times (12%) [12,36,44] and the FV method (Table 6) twice (8%) [13,36]. Thirteen of the 25 studies (52%) compared 1RM predictions performed using a different number of loads [6,12,18,19,30,31,34,36,39–43]. Of the studies examining the use of a different number of loads, four [6,30,34,36] also assessed whether this influenced the reliability of 1RM predictions. Upper body exercises were examined 19 times, whereas lower body exercises were examined 11 times. Fifteen studies examined free-weight exercises, 12 exercised on the Smith machine and three studies examined different machine-based exercises and report valid 1RM predictions in bilateral and unilateral leg extension [29], cable lat pull-down and cable seated row [42] and the pin-loaded chest press and leg press [13]. A further two studies have also directly compared 1RM prediction validity and reliability between Smith machine and free-weight variations of the same movement pattern, specifically for the bench press [17] and bent-over row [15]. Seventeen (68%) studies developed LVRs using MCV exclusively [6,12,13,18,19,28–31,33,34,37,39–43] and two (8%) solely used MPV [17,35]. Additionally, five studies (20%) have directly compared 1RM predictions performed using different velocity metrics; namely the MCV, MPV and PCV [9,15,32,38,44]. Twelve different velocity monitoring devices were used when developing LVRs across the 25 included studies. These devices include two different smartphone applications [19,28,29,42], eight different linear position transducers [6,9,17–19,30,32–34,37–44], one camera-based optoelectronic system [19] and two different inertial measurement units [13,19]. Lastly, five studies examined how the specific loads used to develop the LVR influences 1RM prediction validity and reliability [12,34,36,39,44].

**Table 4:** Summary of results utilising the minimal velocity threshold prediction method.

Study (year)	Device (Make)	Velocity variable	Exercise (modality)	Point method; loads (%1RM)	Validity	Reliability
Balsalobre-Fernández et al.(2018) [28]	Smartphone app (PowerLift)	MCV	Bench press (FW)	4; 75, 80, 85, 90	g = 0.12 (95% CI:−0.75, 1.00), r = 0.98 (90% CI:0.97, 0.99), MAE = 5.5±9.6kg	NS
Balsalobre-Fernández et al.(2019) [29]	Smartphone App (MyLift)	MCV	Leg extension (M)	2; 40, 70	Bilateral: d = 0.02 (95% CI:–0.69, 0.73), r = 0.96, SEE = 3.4kg Dominant: d = –0.25 (95% CI:–1.00, 0.48), r = 0.96, SEE = 2.2kg Non-dominant: d = –0.33 (95% CI:–1.17, 0.51), r = 0.88, SEE = 3.6kg	NS
Balsalobre-Fernández et al. (2021) [37]	LPT (T-Force System; Ergotech)	MCV	Bench press (SM)	NS; 40 to 80%	Isotonic: d = 0.17, r = 0.97 MAE: 3.4±4.4 kg CO: d = 0.24, r = 0.99; MAE: 3.0±3.6 kg	NS
Banyard et al.(2017) [6]	LPT (PT5A-250, Celesco Transducer Products)	MCV	Back Squat (FW)	5; 20, 40, 60, 80, 90 4; 20, 40, 60, 80 3; 20, 40, 60	5; d = 0.71 (95% CI≈0.33, 1.12), r = 0.93, SEE = 10.6kg, CV = 7.4% 4; d≈0.80 (95% CI≈0.42, 1.23), r = 0.87, SEE = 12.9kg, CV = 9.1% 3; d = 1.04(95% CI≈0.67, 1.50), r = 0.78, SEE = 17.2kg, CV = 12.8%	5; d = –0.02, CV = 5.7%, ICC = 0.92, SEM = 8.6kg 4; d = –0.05, CV = 7.2%, ICC = 0.87, SEM = 11.1kg 3; d = –0.05, CV = 12.2%, ICC = 0.72, SEM = 16.8kg
Benavides-Ubric et al. (2020) [38]	LPT (T-Force System; Ergotech)	MCV MPV PCV	Deadlift (FW)	11; 30, 35, 40, 45, 50, 55, 60, 65, 70, 75, 80	MCV: g = 0.14 to 0.19, r = 0.88 to 0.97, SEE = 4.8 to 8.9 kg MPV: g = 0.04 to 0.09, r = 0.90 to 0.97, SEE = 4.4 to 8.2 kg PCV: g = 0.19 to 0.21, r = 0.91 to 0.98, SEE = 4.1 to 8.0 kg	NS
Caven et al. (2020) [43]	LPT (GymAware Powertool; Kinetic Performance Technology)	MCV	Bench press (FW) Squat (FW)	Bench press: 8; 40, 45, 55, 60, 70, 80, 85, 90 2; 40, 90 Squat: 8; 20, 30, 45, 55, 65, 75, 85, 90 2; 20, 90	Bench press: 8; d = 0.10 to 0.11, r = 0.94 to 0.97 2; d = 0.32 to 0.33, r = 0.84 to 0.89 Squat: 8; d = 0.16 to 0.19, r = 0.86 to 0.95 2; d = 0.23 to 0.29, r = 0.76 to 0.93	NS
Fernandes et al. (2021) [39]	LPT (FitroDyne rotary encoder; Fitronic)	MCV	Bench press (SM) Bent-over row (SM)	7; 20, 30, 40, 50, 60, 70, 80 2a; 20, 80 2b; 20, 40 2c; 60, 80	Bench press: 7; d = –0.02, r = 0.8, MAE = –0.4±10.4kg 2a; d = 0.05, r = 0.87, MAE = 1.0±9.6 kg 2b; d = 0.21, r = 0.58, MAE = 4.3±19.6kg 2c; d = 0.08, r = 0.87, MAE = 1.6±9.9kg Bent-over row: 7; d = 0.34, r = 0.77, MAE = 6.4±14.1kg 2a; d = 0.56, r = 0.74, MAE = 10.3±14.5kg 2b; d = –0.14, r = 0.72, MAE = –2.4±20.8kg 2c; d = 0.50, r = 0.77, MAE = 10.1±17.0kg	NS
Garcia-Ramos et al.(2018a) [9]	LPT (T-Force System, Ergotech)	MCV MPV	Bench press CO (SM) Bench press EC (SM)	Bench press CO MCV: 2a*; 37.8, 75.5 MPV: 2b*; 40.4, 76.7 Bench press EC MCV: 2c*; 52.1, 82.7 MPV; 2d*; 55.5, 82.9	2a; d = 0.02, r = 0.957, SB±RE = 0.2±3.7kg, R^2^ = 0.053 2b; d = 0.04, r = 0.956, SB±RE = 0.5±3.8kg, R^2^ = 0.082 2c; d = –0.17, r = 0.976, SB±RE = −2.3±3.1kg, R^2^ = 0.072 2d; d = –0.03, r = 0.977, SB±RE = –0.4±3.0kg, R^2^ = 0.080	2a; d = –0.07, CV = 4.55% (95% CI:3.60, 6.19), ICC = 0.92 (95% CI:0.84, 0.96) 2b; d = –0.03, CV = 5.11% (95% CI:4.04, 6.96), ICC = 0.90 (95% CI:0.79, 0.95) 2c; d = –0.05, CV = 3.16% (95% CI:2.50, 4.30), ICC = 0.95 (95% CI:0.89, 0.97) 2d; d = –0.08, CV = 3.05% (95% CI:2.41, 4.15), ICC = 0.95 (95% CI:0.89, 0.98)
Garcia-Ramos et al.(2019) [30]	LPT (T-Force System, Ergotech)	MCV	Prone row (FW)	4*; 48.9, 60.2, 71.3, 82.2 2*; 48.9, 82.2	4; d = –0.02, r = 0.926, MAPE = –0.27%, SB±RE = 0.24±5.42kg, R^2^ = 0.010 2; d = 0.06, r = 0.926, MAPE = 0.86%, SB±RE = −0.78±5.30kg, R^2^<0.001	4; d = –0.02, CV = 5.19 (95% CI:3.90, 7.79), ICC = 0.90 (95% CI:0.75, 0.96) 2; d = 0.11, CV = 6.89 (95% CI:5.17, 10.33), ICC = 0.81 (95% CI:0.56, 0.93)
Hughes et al.(2019) [36]	LPT (GymAware Powertool; Kinetic Performance Technology)	MCV	Back squat (FW)	5; 20, 40, 60, 80, 90 4a; 20, 40, 60, 80 4b; 40, 60, 80, 90	5; d = −0.37, r = 0.91 to 0.95 4a; d = −0.48, r = 0.91 to 0.95 4b; d = −0.24, r = 0.91 to 0.95	5; d = −0.05 (95% CI:−0.67, 0.57), CV = 5.0% (95% CI:3.9, 7.0), ICC = 0.92 (95% CI:0.82, 0.97) 4a; d = −0.10 (95% CI:−0.71, 0.53), CV = 4.9% (95% CI:3.9, 7.0), ICC = 0.92 (95% CI:0.84, 0.97) 4b; d = −0.01 (95% CI:−0.63, 0.61), CV = 3.6% (95% CI:2.8, 5.1), ICC = 0.96 (95% CI:0.91, 0.98)
Janicijevic et al. (2021) [40]	LPT (T-Force System; Ergotech)	MCV	Bench press (SM)	4; 45, 60, 75, 90 4P; 45, 60, 75, 90 2; 45, 90	4; d = 0.09; r = 0.98 to 0.99; SEE = 2.79±2.29 (4.6 to 5.5%) kg 4P; d = 0.06, r = 0.96 to 0.97, SEE = 3.54±3.31 (≈5.3 to 5.7%) kg 2; d = 0.08, r = 0.99, SEE = 3.09±2.66 (4.7 to 5.5%) kg	NS
Jiménez-Alonso et al.(2020) [31]	LPT (T-Force System, Ergotech)	MCV	Bench press (FW)	4; 40, 55, 70, 85 2; 40, 85	KR: 4; g = –0.13 (95% CI:–0.85, 0.58), r = 0.99 (95% CI:0.97, 1.00), SEE = 2.62kg (95% CI:1.90, 4.22) 2; g = –0.17 (95% CI:–0.88, 0.63), r = 0.99 (95% CI:0.97, 1.00), SEE = 2.52kg (95% CI:1.83, 4.06) CON: 4; g = –0.11 (95% CI:–0.83, 0.60), r = 0.97 (95% CI:0.92, 0.99), SEE = 4.14kg (95% CI:3.00, 6.68) 2; g = –0.09 (95% CI:–0.80, 0.63), r = 0.97 (95% CI:0.91, 0.99), SEE = 4.44kg (95% CI:3.22, 7.16)	NS
Jukic et al.(2020) [18]	LPT (GymAware Powertool; Kinetic Performance Technology)	MCV	Deadlift (FW; with lifting straps [W] and without lifting straps [N])	5; 20, 40, 60, 80, 90 5P; 20, 40, 60, 80, 90 2; 40, 90	Deadlift W: 5; d = 0.37 to 0.40, r = 0.86 to 0.90, SEE = 13.2 to 15.9 kg, SB±RE = −10.8 to −13.2±12.9 to 18.5 kg 5P; d = 0.01, r = 0.80 to 0.83, SEE = 17.0 to 18.5 kg, SB±RE = −0.03 to −0.5±21.0 to 21.8 kg 2; d = 0.36 to 0.40, r = 0.89 to 0.93, SEE = 11.6 to 14.3 kg, SB±RE = −10.9 to −13.1±11.6 to 17.0 kg Deadlift N: 5P; d = 0.07 to 0.08, r = 0.92 to 0.98, SEE = 5.0 to 10.9 kg, SB±RE = −2.0 to −2.5±8.8 to 12.4 kg 5L; d = 0.00 to 0.02, r = 0.93 to 0.96, SEE = 7.5 to 10.1 kg, SB±RE = −0.1 to −0.6±8.5 to 10.0 kg 2; d = 0.11 to 0.12, r = 0.92 to 0.98, SEE = 5.7 to 10.7 kg, SB±RE = −3.3 to −3.4±7.9 to 11.4 kg	NS
Lake et al.(2017) [32]	LPT (Chronojump; Boscosystem)	MPV MAV	Deadlift (FW)	6; 65, 70, 75, 80, 85, 90	MPV-70: g = −0.77 (95% CI:−1.58, 0.08), r = 0.73, MAE = 16.3kg (95% CI:9.8, 22.8) MAV-70: g = −1.20 (95% CI:−2.06, −0.33), r = 0.60, MAE = 25.5kg (95% CI:17.8, 33.1) MPV-80: g = −1.10 (95% CI:−1.96, –0.24), r = 0.84, MAE = 23.8kg (95% CI:18.8, 28.8) MAV-80: g = −1.24 (95% CI:−2.21, −0.37), r = 0.91, MAE = 27.8kg (95% CI:23.8, –31.7)	NS
Loturco et al.(2016) [35]	LPT (T-Force System, Ergotech)	MPV	Half squat (SM)	6*; ~50, 60, 70, 80, 90, >95	d = 0.04 to 0.16, CV = 0.30 to 0.75%, R^2^ = 0.9661	NS
Loturco et al.(2017) [17]	LPT (T-Force System, Ergotech)	MPV	Bench press (FW/SM)	7*; ~40, 50, 60, 70, 80, 90, >95	Bench press (FW): d = 0.05 to 0.10, CV = 0.86 to 1.37%, R^2^ = 0.9551 Bench press (SM): d = 0.01 to 0.07, CV = 0.82 to 1.48%, R^2^ = 0.9697	NS
Loturco et al.(2018) [15]	LPT (T-Force System, Ergotech)	MCV MPV PCV	Bent-over row (FW/SM) Prone row (FW)	8; 30, 40, 50, 60, 70, 80, 90, >95	Bent-over row (SM): MCV; d = 0.21 to 0.47, CV = 3.26 to 3.73%, R^2^ = 0.8902, SEE = 7.14% MPV; d = 0.14 to 0.30, CV = 2.80 to 3.71%, R^2^ = 0.8972, SEE = 6.91% PCV; d = 0.25 to 0.43, CV = 3.66 to 4.06%, R^2^ = 0.8675, SEE = 7.84% Prone row (FW): MCV; d = 0.38 to 0.75, CV = 3.46 to 3.86%, R^2^ = 0.9088, SEE = 6.27% MPV; d = 0.35 to 0.69, CV = 3.09 to 3.67%, R^2^ = 0.9013, SEE = 6.51% PCV; d = 0.53 to 1.10, CV = 3.94 to 4.83%, R^2^ = 0.8997, SEE = 6.56% Bent-over row (FW): MCV; d = 0.28 to 0.48, CV = 3.56 to 4.42%, R^2^ = 0.796, SEE = 10.03% MPV; d = 0.23 to 0.38, CV = 3.60 to 4.11%, R^2^ = 0.799, SEE = 9.96% PCV; d = 0.23 to 0.48, CV = 3.74 to 4.10%, R^2^ = 0.7863, SEE = 10.27%	NS
Pérez-Castilla et al.(2019a) [42]	LPT (Real Power Pro, Globus) Smartphone app (PowerLift)	MCV	Lat pulldown (M) Seated cable row (M)	4*; ~40, 55, 70, 85 2*; ~40, 85	Lat pulldown LPT: 4; g = −0.03, r = 0.97, SEE = 4.51kg, SB±RE = −0.65±4.61kg 2; g = −0.04, r = 0.98, SEE = 4.37kg, SB±RE = −0.80±4.29kg App: 4; g = −0.05, r = 0.98, SEE = 4.29kg, SB±RE = −1.12±5.00kg 2; g = −0.08, r = 0.98, SEE = 4.30kg, SB±RE = −1.75±5.42kg Seated cable row LPT: 4; g = 0.02, r = 0.98, SEE = 3.61kg, SB±RE = 0.44±3.54kg 2; g = 0.00, r = 0.99, SEE = 3.88kg, SB±RE = −0.02±3.79kg App: 4; g = 0.09, r = 0.96, SEE = 5.12kg, SB±RE = 1.64±5.09kg 2; g = 0.06, r = 0.96, SEE = 5.44kg, SB±RE = 1.11±5.36kg	NS
Pérez-Castilla et al.(2019b) [19]	LPT (T-Force System; Chronojump; Speed4Lifts) CBOD (Velowin) IMU (PUSH Band; Beast Sensor) Smartphone App (MyLift)	MCV	Bench Press (SM)	5; 45, 55, 65, 75, 85 2; 45, 85	T-force: 5; g = 0.35 (95% CI:–1.19, 0.49), r = 0.97 (95% CI:0.91, 0.99), SEE = 3.16 (95% CI:2.30, 5.20) 2; g = 0.35 (95% CI:–1.19, 0.49), r = 0.97 (95% CI:0.91, 0.99), SEE = 3.12 (95% CI:2.27, 5.13) Chronojump: 5; g = –0.13 (95% CI:–0.71, 0.97), r = 0.95 (95% CI:0.84, 0.98), SEE = 4.11 (95% CI:3.00, 6.76) 2; g = –0.08 (95% CI:–0.76, 0.91), r = 0.96 (95% CI:0.87, 0.99), SEE = 3.68 (95% CI:2.68, 6.05) Speed4Lifts: 5; g = 0.30 (95% CI:–1.14, 0.54), r = 0.97 (95% CI:0.91, 0.99), SEE = 3.02 (95% CI:2.21, 4.98) 2; g = 0.31 (95% CI:–1.15, 0.53), r = 0.97 (95% CI:0.91, 0.99), SEE = 3.13 (95% CI:2.28, 5.15) Velowin: 5; g = 0.18 (95% CI:–1.02, 0.66), r = 0.97 (95% CI:0.91, 0.99), SEE = 3.15 (95% CI:2.30, 5.18) 2; g = 0.24 (95% CI:–1.08, 0.59), r = 0.97 (95% CI:0.90, 0.99), SEE = 3.27 (95% CI:2.39, 5.38) PUSH Band: 5; g = –0.83 (95% CI:–0.04, 1.70), r = 0.94 (95% CI:0.82, 0.98), SEE = 4.45 (95% CI:3.25, 7.32) 2; g = –0.70 (95% CI:–0.16, 1.57), r = 0.93 (95% CI:0.79, 0.98), SEE = 4.80 (95% CI:3.50, 7.90) Beast Sensor: 5; g = –0.84 (95% CI:–0.04, 1.71), r = 0.68 (95% CI:0.24, 0.89), SEE = 9.44 (95% CI:6.89, 15.5) 2; g = 0.36 (95% CI:–1.20, 0.49), r = 0.50 (95% CI:–0.03, 0.81), SEE = 11.2 (95% CI:8.15, 18.4) My Lift 5; g = 0.37 (95% CI:–1.22, 0.47), r = 0.94 (95% CI:0.82, 0.98), SEE = 4.46 (95% CI:3.25, 7.33) 2; g = 0.40 (95% CI:–1.24, 0.45), r = 0.95 (95% CI:0.84, 0.98), SEE = 4.13 (95% CI:3.02, 6.80)	NS
Pérez-Castilla et al.(2020) [33]	LPT (T-Force System, Ergotech)	MCV	Bench Press (SM)	2*; 46.4, 84.5	Close grip: d = 0.10 to 0.22 (NS), r = 0.98, SEE = 3.0kg, R^2^ = 0.04, SB±RE = –3.0±3.0kg Medium grip: d = 0.10 to 0.22 (NS), r = 0.97, SEE = 3.0kg, R^2^ = 0.03, SB±RE = –2.0±3.0kg Wide grip: d = 0.10 to 0.22 (NS), r = 0.98, SEE = 3.0kg, R^2^ = 0.02, SB±RE = –1.0±3.0kg Self-selected grip: d = 0.10 to 0.22 (NS), r = 0.96, SEE = 4.0kg, R^2^ = 0.00, SB±RE = –2.0±4.0kg	NS
Ruf et al.(2018) [34]	LPT (GymAware Powertool; Kinetic Performance Technology)	MCV	Deadlift (FW)	3a; 20, 40, 60 3b; 40, 60, 80 3c; 60, 80, 90 4a; 20, 40, 60, 80 4b; 40, 60, 80, 90 5; 20, 40, 60, 80, 90	3a; g = –0.31 to –0.27,r = –0.96 (95% CI:–0.98, –0.93) 3b; g = –0.28 to –0.14, r = –0.96 (95% CI:–0.98, –0.94) 3c; g = –0.14 to –0.13, r = –0.93 (95% CI:–0.96, –0.88) 4a; g = –0.25 to –0.20, r = –0.98 (95% CI:–0.99, –0.96) 4b; g = –0.19 to –0.13, r = –0.97 (95% CI:–0.98, –0.95) 5; g = –0.19 to –0.17, r = –0.98 (95% CI:–0.99, –0.97)	3a; g = –0.02 (95% CI:–0.86, 0.82), CV = 1.9 to 4.4% (NS), ICC = 0.95 to 0.997 (NS), MAE = 3.4 to 7.5kg (NS) 3b; g = 0.16 (95% CI:–0.68, 0.99), CV = 1.9 to 4.4% (NS), ICC = 0.95 to 0.997 (NS), MAE = 3.4 to 7.5kg (NS) 3c; g = 0.02 (95% CI:–0.81, 0.86), CV = 1.9 to 4.4% (NS), ICC = 0.95 to 0.997 (NS), MAE = 3.4 to 7.5kg (NS) 4a; g = 0.07 (95% CI:–0.77, 0.90), CV = 1.9 to 4.4% (NS), ICC = 0.95 to 0.997 (NS), MAE = 3.4 to 7.5kg (NS) 4b; g = 0.06 (95% CI:–0.77, 0.90), CV = 1.9 to 4.4% (NS), ICC = 0.95 to 0.997 (NS), MAE = 3.4 to 7.5kg (NS) 5; g = 0.03 (95% CI:–0.80, 0.87), CV = 1.9 to 4.4% (NS), ICC = 0.95 to 0.997 (NS), MAE = 3.4 to 7.5kg (NS)
Thompson et al. (2021) [41]	LPT (GymAware Powertool; Kinetic Performance Technology)	MCV	Back Squat (FW)	4; 0, 30, 50, 80 4P; 0, 30, 50, 80 7; 0, 30, 40, 50, 60, 70, 80 7P; 0, 30, 40, 50, 60, 70, 80	4; d = 0.12 (95% CI:–0.66, 0.90), r = 0.99, SEE = 3.26 kg 4P; d = –0.06 (95% CI:–0.82, 0.74), r = 0.98, SEE = 1.82 kg 7; d = 0.19 (95% CI:–0.59; 0.97), r = 0.99, SEE = 3.11 kg 7P; d = 0.04 (95% CI:–0.74, 0.81), r = 0.98, SEE = 4.06 kg	NS

*Note*: Linear regression models were used unless noted otherwise. **1RM =** one repetition maximum, **CBOD** = camera-based optoelectronic device, **CI =** confidence interval, **CO =** concentric phase only, **CON =** no verbal velocity performance feedback, **CV =** coefficient of variation, **EC =** eccentric to concentric phase, **FW =** free-weight, **ICC =** intraclass correlation coefficient, **IMU** = inertial measurement units, **KR =** verbal velocity performance feedback, **LPT =** linear position transducer, **M =** machine, **MAE =** mean absolute error, **MAPE =** mean absolute percentage error, **MAV =** mean acceleration phase velocity, **MCV** = mean concentric velocity, **MPV =** mean propulsive velocity, **N =** deadlift without lifting straps, **NS =** not specified, **P =** polynomial regression model, **PV =** peak velocity, **SB±RE** = systematic bias ± random error, **SEE =** standard error of the estimate, **SEM =** standard error of the measurement, **SM =** Smith machine, **W =** deadlift with lifting straps. *Individualised loads.

**Table 5:** Summary of results utilising the load at zero velocity prediction method.

Study (year)	Device (Make)	Velocity variable	Exercise (modality)	Point method; loads (%1RM)	Validity	Reliability
Hughes et al.(2019) [36]	LPT (GymAware Powertool; Kinetic Performance Technology)	MCV	Back squat (FW)	5; 20, 40, 60, 80, 90 4a; 20, 40, 60, 80 4b; 40, 60, 80, 90	5; d = 0.01, r = NS 4a; d = 0.01, r = –0.50 4b; d = 0.04, r = NS	5; d = 0.05 (95% CI:–0.57, 0.67), CV = 8.2% (95% CI:6.4, 11.6), ICC = 0.82 (95% CI:0.63, 0.90) 4a; d = –0.03 (95% CI:–0.65, 0.59), CV = 8.5% (95% CI:6.6, 12.1), ICC = 0.78 (95% CI:0.57, 0.90) 4b; d = 0.10 (95% CI:–0.52, 0.72), CV = 8.6% (95% CI:6.7, 12.3), ICC = 0.81 (95% CI:0.62, 0.91)
Jidovtseff et al.(2011) [12]	LPT (PT5DC, Celesco Transducer Products)	MCV	Bench Press (SM)	4a**; 35, 50, 70, 90 4b**; 30, 50, 70, 95 3**; 40, 60, 80 2a**; 30 to 35, 70 2b**; 40, 60	Pooled†: d = –0.49 (95% CI: –0.11, –0.75); SEE = ≤5.0kg 4a; r = 0.96 4b; r = 0.95 3; r = 0.95 2a; r = 0.96 2b; r = 0.96	NS
Sayers et al.(2018) [44]	LPT (WS17KT, ASM)	MCV PCV	Bench press throw (SM)	3a; 30, 40, 50 3b; 40, 50, 60 3c; 50, 60, 70	MCV: 3a; ICC = 0.868(95% CI:0.558, 0.966), R^2^ = 0.96, SEE = 4.4kg 3b; ICC = 0.855(95% CI:0.521, 0.962), R^2^ = 0.52, SEE = 18.9kg 3c; ICC = 0.849(95% CI:0.506, 0.960), R^2^ = 0.78, SEE = 11.0kg PCV: 3a; ICC = 0.967(95% CI:0.890, 0.990), R^2^ = 0.87, SEE = 10.4kg 3b; ICC = 0.680(95% CI:0.204, 0.896), R^2^ = 0.89, SEE = 10.5kg 3c; ICC = 0.867(95% CI:0.604, 0.960), R^2^ = 0.85, SEE = 11.6kg	NS

*Note*: **1RM =** one repetition maximum, **CI =** confidence interval, **ICC =** intraclass correlation coefficient, **LPT =** linear position transducer, **MCV** = mean concentric velocity, **NS =** not specified, **PCV =** peak concentric velocity, **SEE** = standard error of estimate, **SM =** Smith machine. **Independent samples for models 4a/2a, 4b/2a, 3/2b. †Exact effect size and SEE values per prediction model are unspecified.

**Table 6:** Summary of results utilising the force velocity prediction method.

Study (year)	Device (Make)	Velocity variable	Exercise (modality)	Point method; loads (%1RM)	Validity	Reliability
Hughes et al.(2019) [36]	LPT (GymAware Powertool; Kinetic Performance Technology)	MCV	Back squat (FW)	5; 20, 40, 60, 80, 90 4a; 20, 40, 60, 80 4b; 40, 60, 80, 90	5; d = 0.33, r = 0.91 to 0.95 4a; d = –0.09, r = 0.91 to 0.95 4b; d = –0.08, r = 0.91 to 0.95	5; d = 0.33 (95% CI:–0.30, 0.95), ICC = 0.00 (95% CI:–0.39, 0.39) 4a; d = –0.07 (95% CI:–0.69, 0.55), ICC = –0.28 (95% CI:–0.60, 0.12) 4b; d = 0.50 (95% CI:–0.14, 1.12), ICC = –0.11 (95% CI:–0.48, 0.29)
Picerno et al.(2016) [13]	Triaxial inertia sensor (Sensorize)	MCV	Chest press CO (M) Leg press CO (M)	3; 50, 65, 80	Chest press: d = –0.05 (95% CI:–0.50, 0.41), r = 0.99, SEE = 1.2kg, MAE = 1.4kg, MAPE = 1.5%, Bias = –1.32 (95% CI:–3.58, 0.94) Leg press: d = –0.03 (95% CI:–0.49, 0.43), r = 0.99, SEE = 2.1kg, MAE = 1.8kg, MAPE = 0.8%, Bias = –1.76 (95% CI:–5.81, 2.29)	NS

*Note*: **1RM =** one repetition maximum, **CI =** confidence interval, **CO =** concentric phase only, **M =** machine, **MAE =** mean absolute error, **MAPE** = mean absolute percentage error, **MCV** = mean concentric velocity, **NS =** not specified, **SEE** = standard error of estimate.

## Discussion

This systematic review aimed to identify key factors which influence the validity of predicting 1RM using LVRs, and the reliability of predictions as a secondary outcome. Based on the evidence available, we identified four primary factors relevant to the efficacy of using these 1RM predictions in practice: i) the number of loads used, ii) the exercise examined, iii), the velocity metric used, and iv) the velocity monitoring device used. In addition to these, four secondary factors were identified which may require further consideration: v) the specific loads used, vi) the provision of velocity feedback, vii) use of lifting straps, and viii) the regression model used. The influence of each of these factors is outlined in the ensuing sections, followed by a summary of practical recommendations for practitioners and researchers to guide best practice and continued development in this field.

### Number of loads

**Fig 3:**
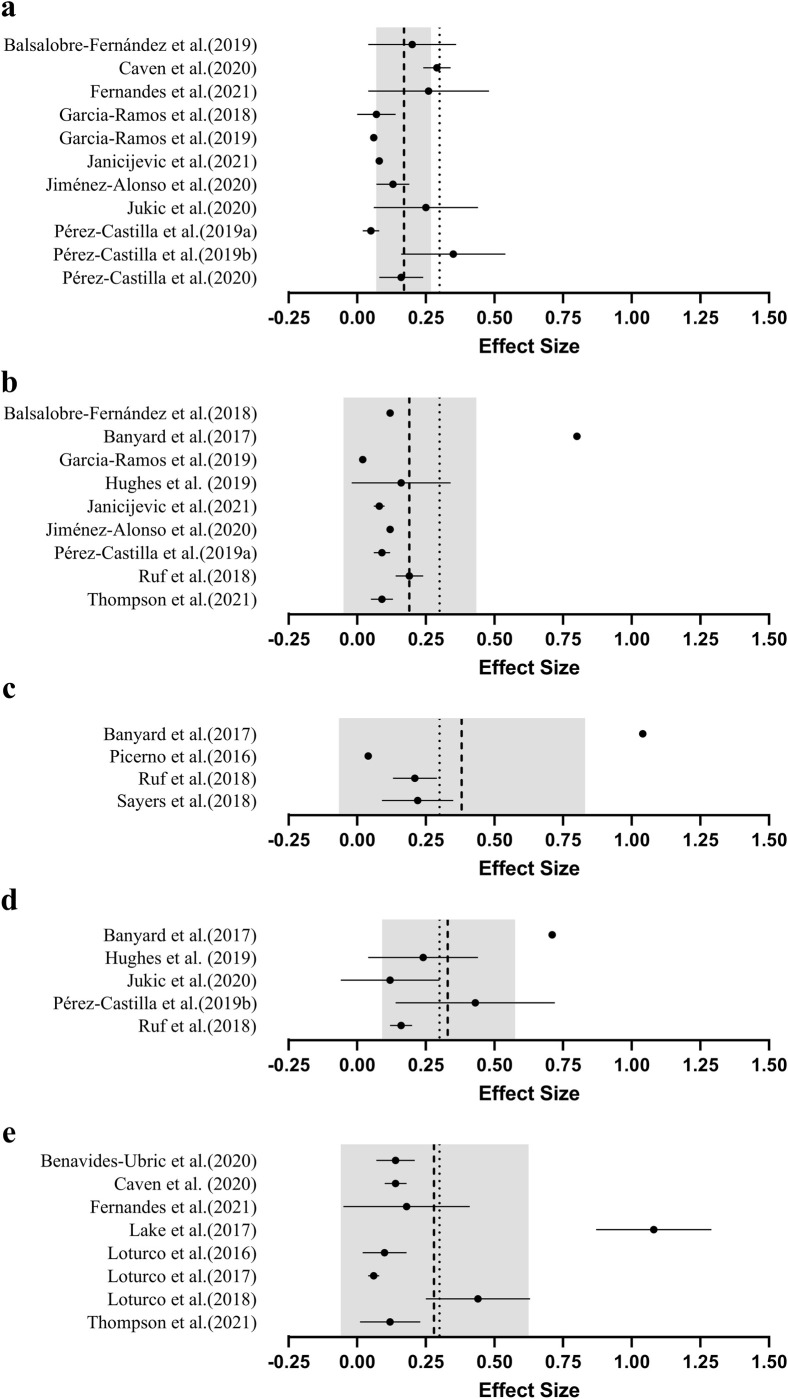
Mean (●) effect sizes (±SD), grouped average (---) and recommended maximum effect size (∙∙∙) for magnitude of difference between predicted and actual 1RM by the number of loads used for their prediction model; a.) 2-point method, b.) 3-point method, c.) 4-point method, d.) 5-point method, e.) ≥6-point method. The grey band represents the standard deviation of the pooled effect sizes. *Note*: Effect sizes closer to zero indicate greater validity (i.e., smaller difference between actual and predicted 1RM).

Maximising the number of different loads used to develop a LVR may enhance the validity of 1RM predictions (Fig 3). However, given that each load used increases the time needed to perform these predictions, developing LVRs from fewer separate loads may be more practical in a real-world setting [6,9,10]. Only two of 13 studies show evidence of improved validity with a greater number of loads [6,43]. For example, a 3-load prediction method overestimates predicted 1RM back squat (i.e., +29.6 kg) to a greater degree (*p* ≤ 0.05) than a 5-load prediction (i.e., +19.4 kg) [6]. Despite this, neither prediction method returned a valid 1RM prediction when using previously determined validity criteria (i.e., ES < 0.30, near-perfect correlation coefficients and low absolute error below 5 kg) [18,19]. Caven and associates reported larger effect sizes using 2-load predictions of bench press (ES = 0.32 to 0.33) and squat (ES = 0.23 to 0.29) 1RM when compared to a model using 8-loads (bench press ES = 0.10 to 0.11; squat ES = 0.16 to 0.19) [43]. The remaining studies highlighted no differences between predictions performed using 2- vs. 4-load [30,31,40,42], 2- vs. 5-load [18,19], 2- vs. 7-load [39], 4- vs. 7-load [41], 4- vs. 7-load [36], 2- vs. 3- vs. 4-load [12], or 3- vs.4- vs. 5-load models [34]. Despite minimal evidence that validity differs between models, the mean grouped effect size appears to increase (i.e., poorer validity) when a greater number of loads are used. Indeed, the mean of studies using 2-load predictions (ES = 0.17, Fig 3A) is within recommended validity (i.e., ES <0.30), while the recommendation is exceeded when 5 or more loads are used to develop a LVR (Fig 3D and 3E) [18]. This may be due to the large effect sizes (ES = 071 to 1.08) reported by two studies using 5 or more loads [6,32].

Although not statistically significant, a greater number of loads is associated with higher reliability and lower variability (ICC = 0.90, CV = 5.19%) when compared to 1RM predictions using fewer loads (ICC = 0.81, CV = 6.89%) [6,30]. However, it should be noted that the test-retest reliability associated with LVR-based 1RM predictions for two of these investigations was lower (i.e., ICC = 0.97 direct 1RM, ICC = 0.90 predicted 1RM) and variability higher (i.e., CV = 2.1 to 2.4% direct 1RM, CV = 5.2 to 5.7% predicted 1RM) than direct assessment, even when the maximal possible number of loads was used [6,30]. Practitioners should therefore be aware that LVR-based 1RM predictions are unlikely to be able to detect small changes in maximal strength (e.g., less than 5 to 7%) that may be measured through direct 1RM assessment. This is a particularly important consideration for athletic cohorts, where smaller changes in maximal strength are common when compared with less trained populations [45].

### Exercise examined

**Fig 4:**
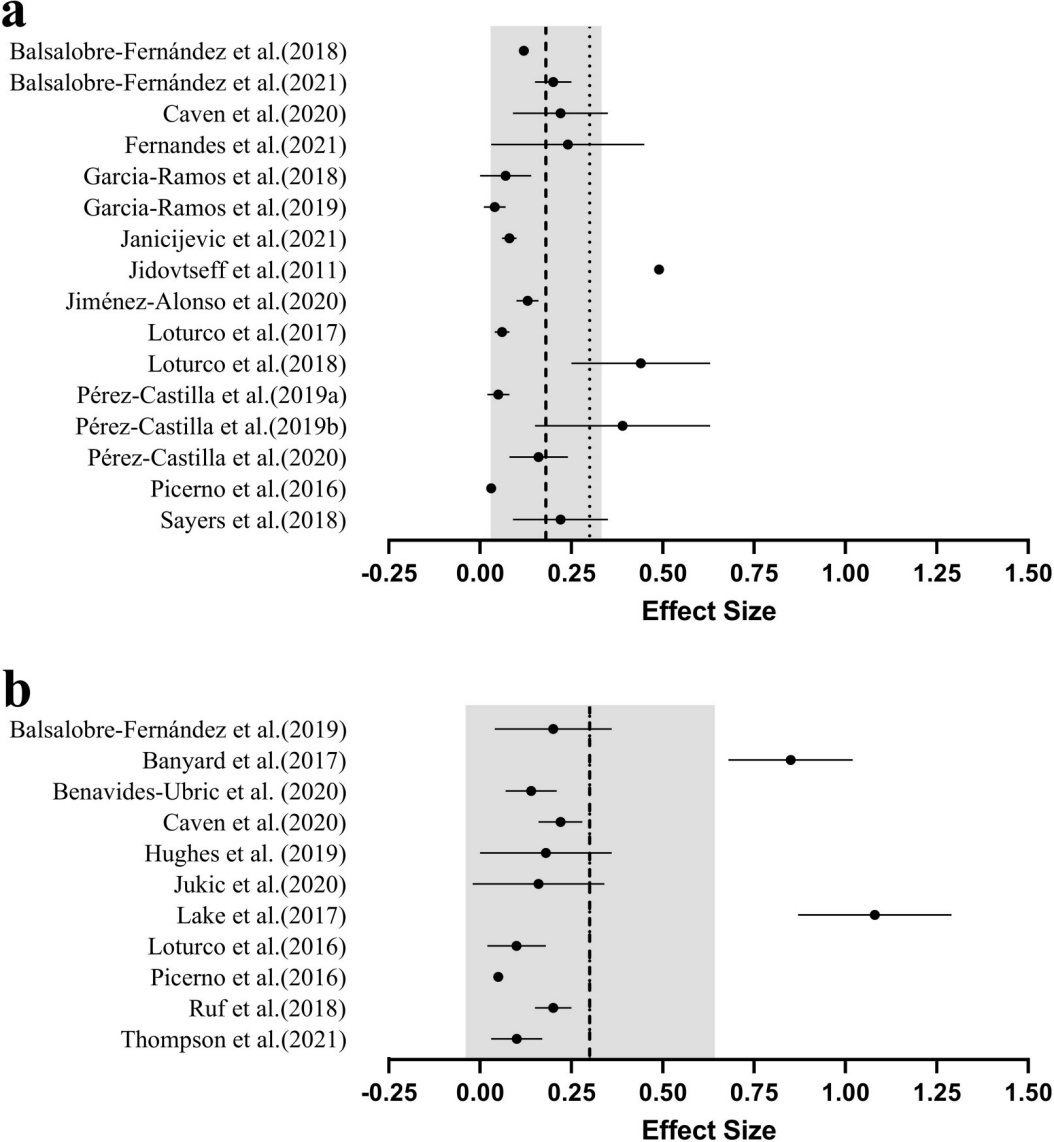
Mean (●) effect sizes (±SD), grouped average (---) and recommended maximum effect size (∙∙∙) for difference between predicted and actual 1RM by exercise group examined; a.) upper body exercises, b.) lower body exercises. The grey band represents the standard deviation of the pooled effect sizes. *Note*: Effect sizes closer to zero indicate greater validity (i.e., smaller difference between actual and predicted 1RM).

Between-study differences in testing protocols, populations and validity criteria make it difficult to draw strong conclusions regarding the influence of the exercise examined. Indeed, the mean effect size for lower body exercises appears greater (ES = 0.30) relative to upper body exercises (ES = 0.18) but are likely inflated by the large effect sizes reported by studies examining the free-weight back squat and deadlift [6,32] (Fig 4). Free-weight back squats and deadlifts are complex exercises. Outside of technical proficiency, exercise technique can certainly vary at the individual level due to factors as unavoidable as anthropometry (e.g., sitting height, total height, segment length) [46,47]. Whilst further research is required, given the lower validity already reported [6,32,36] it is possible that greater technical demand and individual technical variation of the free-weight back squat and deadlift may impact on the ability to predict 1RM through the LVR. In contrast, Benavides-Ubric and colleagues reported higher validity for the free-weight deadlift and a negligible magnitude of difference (ES = 0.04 to 0.09) to direct 1RM when developing the LVR model across 11 loads [38]. Although currently unknown, it is possible that by recruiting stronger participants (relative deadlift = 2.1 kg∙mass^-1^) than Benavides-Ubric et al. (relative deadlift = 1.8 kg∙mass^-1^), Lake et al. impacted on the ability to predict 1RM from the LVR because lifting velocity is slower in stronger individuals [48]. However, further research is needed to establish the impact of existing strength levels on 1RM prediction using LVRs.

Using a range of other exercises, similar 1RM prediction validity has been reported between exercises [13,15,29,42,43]. For Smith machine exercise, however, Fernandes and associates reported poor validity and high error for both the bench press and, more significantly, the bent-over row. The authors suggest that the particularly high error (i.e., 8.6 to 19.9%) of bent-over row 1RM predictions may be because the reference value used for MVT may have been too slow, and recommend implementing population specific MVT values rather than relying on reference values [39]. In free-weight exercises, Loturco et al. [15] compared validity of different free-weight prone row and bent-over row exercises, and in agreement with work conducted using machine-based exercise, reported comparable validity of LVR-based 1RM predictions between exercises. While additional trunk movement in the bent-over row was hypothesised to somewhat impair the validity of 1RM estimates compared with the more supported prone row, valid 1RM predictions were noted for both exercises. Likewise, comparable effect sizes and correlation coefficients are reported in free-weight bench press and squat with trends remaining consistent across multiple loading models (i.e., 2- vs 8-load models) [43]. These findings are encouraging for practitioners who aim to estimate 1RM from free-weight exercises based on the LVR.

When comparing prediction validity between Smith machine and free-weight variations of the same movement pattern, differences in validity were not observed for the bench press or bent-over row exercises [15]. To date, there are currently no studies available that compare the prediction reliability and validity between free-weight and Smith machine for lower body exercises. Barbell squatting movements, for instance, are complex free-weight exercises with many potential kinematic sources of variation (e.g., spine, ankle, knee and/or hip) [49]. When performed on a Smith machine, the reduced balance demands are theorised to contribute to a greater squat 1RM (i.e., 3.7%) when compared to free-weight squat 1RM (Smith machine: 129.0 kg, free-weight: 124.3 kg) [50]. This is despite greater electromyographic activity of the vastus medialis (49%), gastrocnemius (34%) and biceps femoris (26%) is observed during free-weight squats when compared to Smith machine squats [51]. Taken together, it remains unknown whether Smith machine predictions are transferrable to free-weights for lower body movements. Given a substantial number of studies examining LVRs have been conducted using a Smith machine [9,12,19,33,44], further research is essential to determine transferability of Smith machine predictions.

### Velocity metric

**Fig 5:**
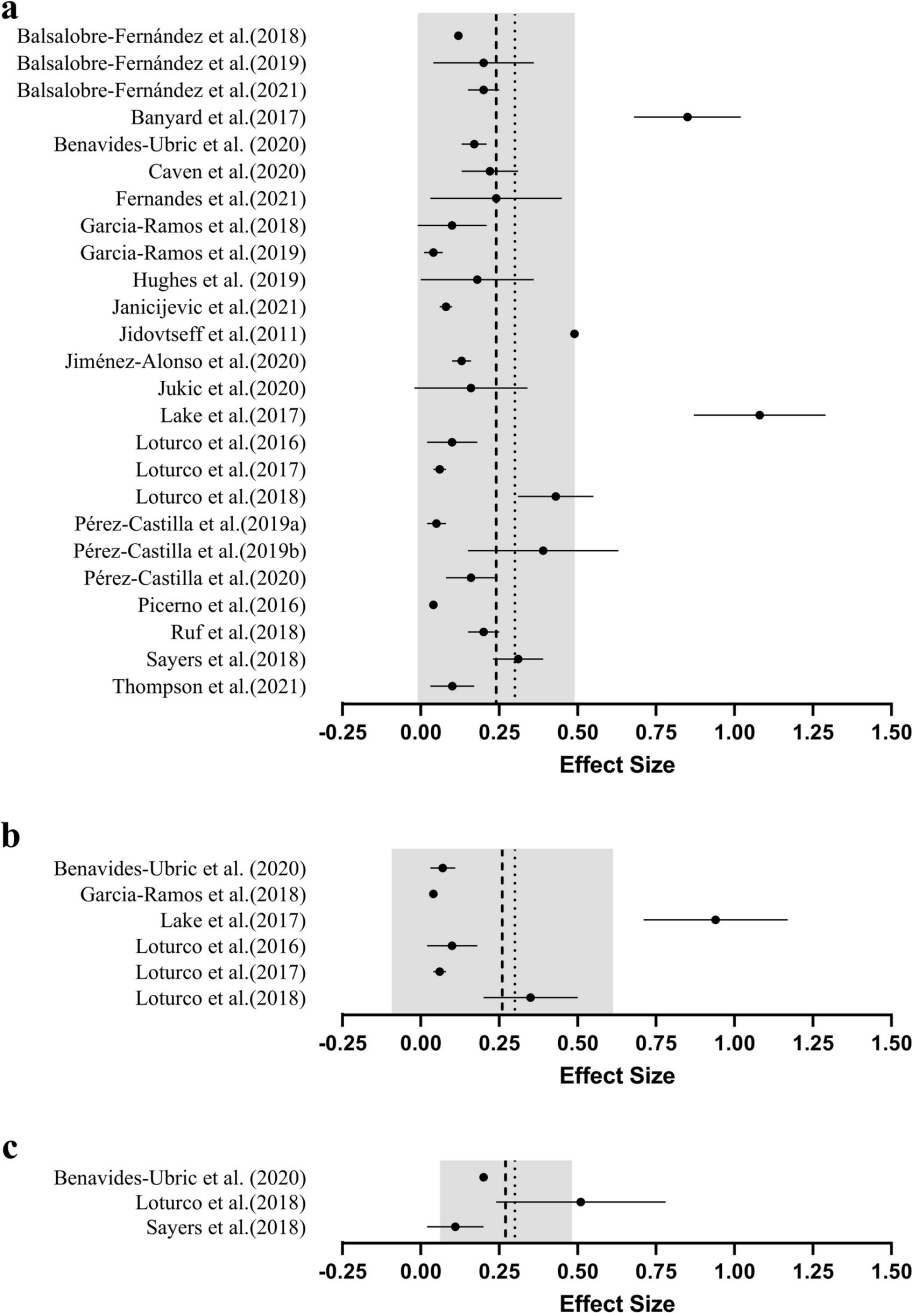
Mean (●) effect sizes (±SD), grouped average (---) and recommended maximum effect size (∙∙∙) for magnitude of difference between predicted and actual 1RM by velocity metric used for maximal strength prediction; a.) mean concentric velocity, b.) mean propulsive velocity, c.) peak concentric velocity. The grey band represents the standard deviation of the pooled effect sizes. *Note*: Effect sizes closer to zero indicate greater validity (i.e., smaller difference between actual and predicted 1RM).

The specific velocity metric used to develop LVRs may be another consideration for practitioners attempting 1RM predictions [14] (Fig 5).García-Ramos et al. [9] reported minimal and likely trivial differences in the validity and reliability of 1RM predictions made using the MCV compared with the MPV, for concentric-only and eccentric-concentric Smith machine bench press. Negligible between-metric differences were also observed when 1RM predictions were performed using MCV, MPV and PCV for the free-weight and Smith machine bent-over row and the free-weight prone row [15]. In contrast to these findings, Sayers et al. [44] observed more valid 1RM predictions when using PCV (R^2^ = 0.85 to 0.89, SEE = 10.5 to 11.6 kg) when compared to MCV (R^2^ = 0.52 to 0.96, SEE = 4.4 to 18.9 kg). It should be noted though that this study predicted bench press 1RM from the LVR of the bench throw, a ballistic exercise that may better suit the use of PCV than MCV [11], and so these results were not surprising. Although it is unclear whether it was included in their final analyses, a large outlier (i.e., actual 1RM ≈ 130 kg, predicted 1RM ≈ 75 kg) in the 3-load model using MCV would undoubtedly impact on the interpretation of validity in this study [44]. Furthermore, no mean differences were observed between measured and predicted 1RM when calculated from MCV, yet differences were observed when predicting 1RM using PCV [44]. Additionally, the Bland-Altman plots generally highlighted wider limits of agreement for predictions performed using PCV than MCV [44]. Therefore, the conflicting results presented by this study may be due to the methodology implemented (i.e., using the bench throw to predict bench press 1RM), and may not be relevant for estimating 1RM strength from non-ballistic exercises.

Lake et al. [32] highlighted improved 1RM prediction validity and reliability when using MPV compared with mean accelerative velocity (i.e., a velocity metric that to the best of our knowledge has never previously been examined). This study defined the MPV metric as the mean velocity achieved “between the first positive velocity to peak displacement” of the deadlift finish position [32]. Since this definition describes the entire concentric phase, rather than just the propulsive phase of movement, this may in fact represent the MPV. Unfortunately, this makes it difficult to draw conclusions from this study with respect to the velocity metric resulting in improved 1RM predictions.

### Device used

**Fig 6:**
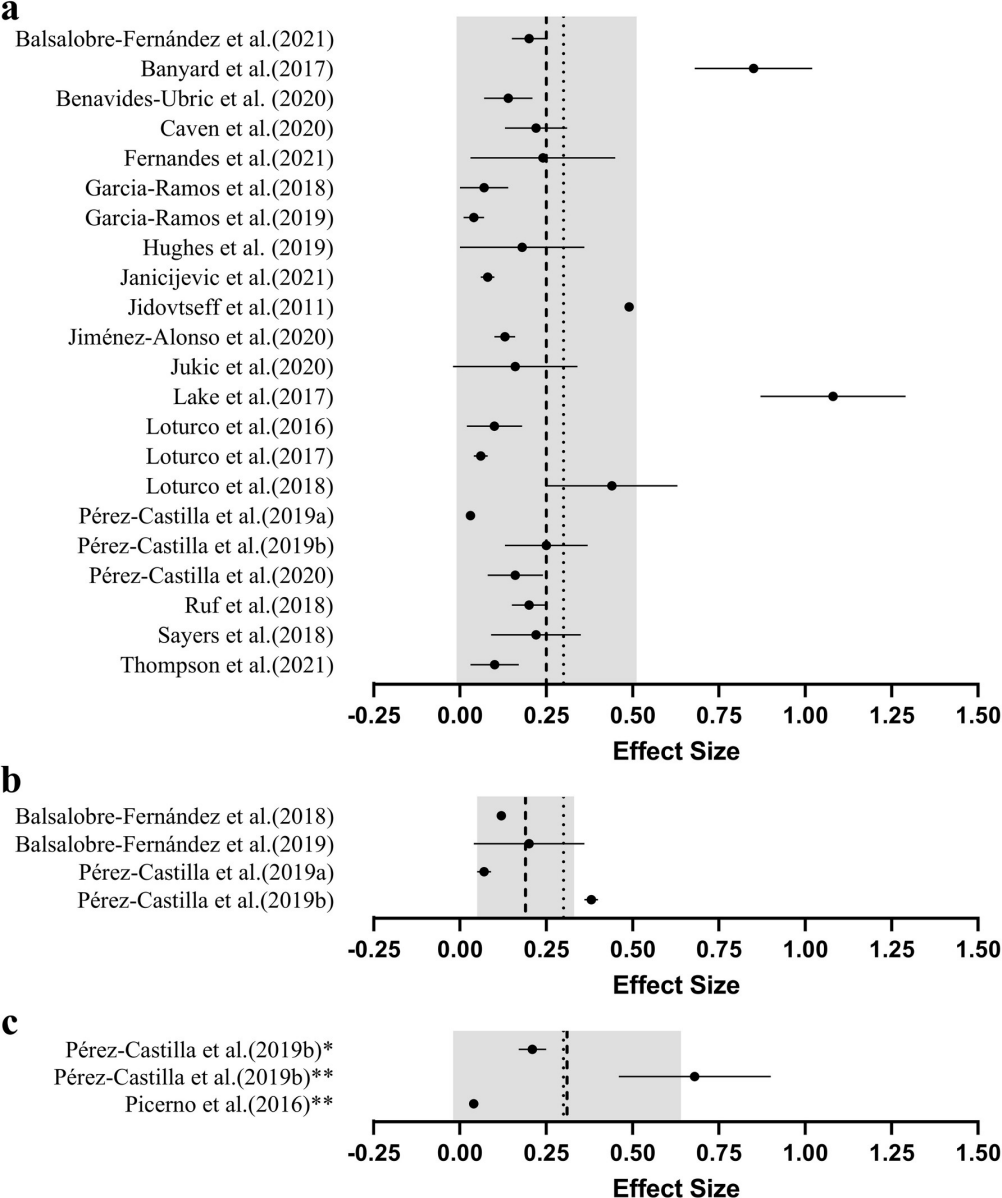
Mean (●) effect sizes (±SD), grouped average (---) and recommended maximum effect size (∙∙∙) for magnitude of difference between predicted and actual 1RM by the velocity monitoring device used; a.) linear position transducer, b.) smartphone application, c.) other devices. *camera-based optoelectronic system, **inertial measurement units. The grey band represents the standard deviation of the pooled effect sizes. *Note*: Effect sizes closer to zero indicate greater validity (i.e., smaller difference between actual and predicted 1RM).

Using valid and reliable tools to quantify repetition velocity is a key consideration for practitioners (Fig 6). A recent systematic review has shown that linear position transducers generally demonstrate the greatest validity and reliability, and are typically favoured over other types of device for monitoring repetition velocity [52]. Given the small but meaningfully systematic differences in velocity previously reported when different linear position transducers are used concurrently [53,54], practitioners should avoid using these devices interchangeably [52].

While several studies have examined between-device differences in the validity and reliability of quantifying velocity metrics, this systematic review identified only two that have examined the influence of the device on the validity of predicting 1RM from LVRs [19,42]. These studies indicate that 1RM predictions calculated from LVRs developed using linear position transducers are no more valid or reliable than those which use a camera-based optoelectrical system (Velowin, Deportec, Murcia, Spain) [19] or a far more cost-effective smartphone application (Powerlift Application; Apple Inc., USA) [19,42]. Further, while no differences were observed between T-force (Ergotech, Murcia, Spain), Chronojump (Boscosystems, Barcelona, Spain) and Speed4lifts (Speed4lifts, Madrid, Spain) linear position transducers, inertial measurement units (PUSH Band, PUSH Inc., Toronto, Canada and Beast Sensor, Beast Technologies, Brescia, Italy) resulted in a potential reduction in 1RM prediction validity (i.e., direct 1RM = 83.8 kg, PUSH Band predicted 1RM = 74.4 to 76.2 kg, Beast Sensor predicted 1RM = 73.4 to 90.0 kg) [19].

In agreement with the current review, previous research has proposed that the exercise examined likely impacts the validity and reliability of velocity measures obtained using different devices, theorising that some devices may be more sensitive to movement outside of a singular plane than others [52]. Indeed, we speculate that the large kinematic variations of more complex exercises likely contribute to greater error in 1RM prediction, error which may be further compounded with varied non-linear sensitivity of velocity monitoring devices. The two studies examining the impact of the device used on LVR 1RM predictions examined the highly controlled lat pulldown [42], seated row [42] and Smith machine bench press [19] exercises. Therefore, it is important to acknowledge that larger between device differences may be observed for other exercises [52], particularly more commonly used free-weight exercise.

### Further considerations

#### Load selection

Although we acknowledge that the specific loads lifted to develop a LVR impact on the reliability and validity of the prediction, a lack of homogeneity in terms of loads used for 1RM prediction make it difficult to draw conclusions. An earlier study concluded that it is unnecessary to use heavier loads (<75% of 1RM) if the difference in velocity between the lightest and heaviest loads is at least 0.5m·s^-1^ [12]. Despite reporting similar correlation coefficients (i.e., r ≥ 0.95), there was no between model comparison on the validity of 4-load (30%, 50%, 70% and 90% 1RM and 35%, 50%, 70% and 90% 1RM) and 2-load models (30–35% and 70% 1RM and 40% and 60% 1RM). Whilst it is currently suggested that a wide range of loads (and thus velocities) should be used [6,10], using heavy loads may be more important than previously suggested. The use of heavy loads would appear intuitive, as it is likely that loads closer to 100% of 1RM would contribute less error than lighter loads when predicting maximal strength through LVRs [18]. Ruf and associates enhanced 1RM prediction validity and reliability by using heavier loads [34] and suggest the use of heavy loads better represents the individual’s maximal strength capabilities. Lighter loads (i.e., 20% 1RM), on the other hand, may achieve velocities high enough to impair limb coordination and result in a more varied muscle activation patterns [55,56]. Indeed, Fernandes et al. observed significantly poorer (p < 0.02) bench press prediction validity (ES = 0.21, r = 0.58) using loads at 20 and 40% of 1RM which was improved markedly (ES = 0.05, r = 0.87) when substituting in a heavy load (i.e., 80% of 1RM) [39]. Further, estimation error for the bench press was greatest when using low-load methods (e.g., 14.2 to 20.4%). Taken together, it appears likely that the combined use of both heavy and light loads contributes to greater 1RM prediction validity and reliability.

#### Velocity feedback

One of the most recent studies in this review examining the free-weight bench press demonstrated that velocity feedback might also influence LVR 1RM prediction validity [31]. Instantaneous feedback regarding repetition velocity resulted in less error (SEE ≈ 2.57kg) when developing LVRs when compared to a non-feedback condition (SEE ≈ 4.29kg). The improved prediction validity was attributed to the greater reliability of repetition velocity observed of the lightest load included in the LVR (40% 1RM). However, given feedback had a trivial impact on the velocity of heavier loads (i.e., 55%, 70% and 85% 1RM) in this study, the provision of feedback may be most important when light loads are included; further supporting the use of heavier loads when developing LVRs (i.e., requiring less attention to the velocity of the lift on behalf of the practitioner). Since similar increases in repetition velocity have been noted for the free-weight squat with heavier loads (~70% 1RM) when feedback was provided [57], the beneficial impacts of verbal velocity feedback may differ slightly between exercises. While future research may aim to determine the influence of this feedback of LVR based 1RM predictions performed for other exercises, practitioners should aim to provide velocity feedback regardless given the documented benefits to both resistance training performance [57,58] and adaptations [59].

**Lifting straps.** One study in this systematic review also investigated the use of lifting straps during LVR-based 1RM predictions in the deadlift exercise [18], which was found to decrease 1RM prediction validity. The authors attributed this finding to an increased difference (i.e., 0.12 m∙s^-1^) between the velocity of the heaviest load used (i.e., velocity of 90% 1RM) and the velocity of 1RM when lifting straps are used when compared to no straps [18]. However, this study concluded LVR 1RM predictions are not valid for the deadlift exercise performed either with or without lifting straps. Practitioners should consider that these findings may transfer to exercises such as the prone and bent-over row for which 1RM prediction may be valid [15,30], particularly since lifting straps are commonly used to bypass the limiting influence of grip strength on the load and volume which can be lifted [60].

**Linear versus polynomial regressions.** Several studies have administered both linear and polynomial regression models for the identical number of loads [18,40,41] with little consensus on the ideal model to use (Table 4). For the deadlift, Jukic et al.[18] suggest that linear regression models may provide more valid 1RM estimates when compared to polynomial models. However, as already stated, none of the models in this study predicted deadlift 1RM to an acceptable degree, with error up to 18.5kg [18]. Significantly greater bench press validity has been reported when using 4-load linear regressions (SEE = 2.79 ± 2.29 kg) when compared to polynomial regression models (SEE = 3.54 ± 3.31 kg); albeit the relative standard error (linear = 4.6 to 5.4%; polynomial ≈ 5.3 to 5.7%) was marginal between models overall [40]. In contrast, 4- and 7-load linear regression models were determined elsewhere to underestimate back squat 1RM when compared to quadratic polynomial models [41]. This may be due to the exercise selection; the Smith machine was used previously by Janicijevic et al.[40], whereas Thompson et al. [41] examined the back squat using free-weights. The somewhat movement-limited nature of the Smith machine yields more reliable velocity data and may lend itself to a linear regression model, whereas polynomial regression models may be more appropriate to account for reduced linearity introduced by complex free-weight movements such as the back squat [41,61,62]. Therefore, when deciding on a regression model approach, the exercise selection should be an important consideration. Practitioners should also be aware that non-linear regressions such as a polynomial model are largely impacted by data outliers, [63] which may be a complication in studies with smaller sample sizes whereby true outlier detection can be difficult.

## Conclusions

This systematic review explored the factors which have previously been theorised to impact the validity and reliability of predicting 1RM from LVRs. The number of loads used to develop a LVR prediction, the exercise tested, velocity metric assessed, and the velocity monitoring device used are likely key factors that contribute to prediction validity and/or reliability. Further factors may require consideration, such as the specific loads used (e.g., %1RM), the provision of feedback on exercise velocity, use of lifting straps for select exercises and the regression models used for estimating 1RM. Following the recommendations outlined above, we suggest that practitioners can likely predict 1RM with acceptable validity and reliability for several common resistance exercises: the Smith machine squat and half squat, the free-weight and Smith machine bench press, bent-over row and prone row, and the pin-loaded lat pulldown, leg extension (bilateral and unilateral), leg press and chest press exercises. Lower validity and reliability may be observed when predicting maximal strength in more complex free-weight exercises such as the barbell back squat and deadlift. Lastly, we acknowledge that much of the available literature has only recruited male participants; thus, we can only extrapolate these findings to female subjects until such time that more research is conducted with females.

### Practical applications

**Fig 7:**
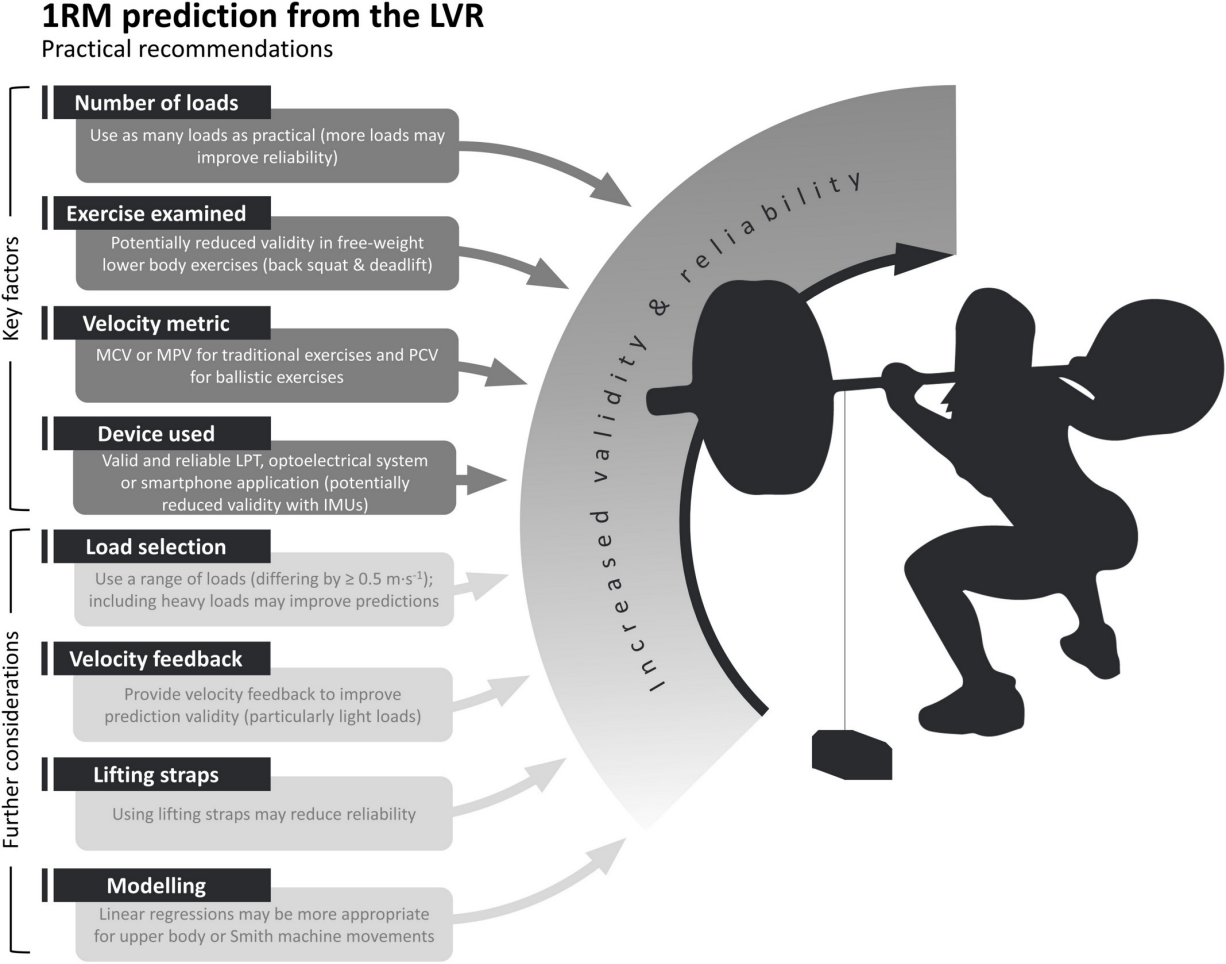
Summary of the practical recommendations for use of a LVR to predict 1RM.

Although the ideal number of loads used for 1RM prediction remains unknown, we suggest that LVRs should be developed using as many loads as practically possible (Fig 7). Comparable validity has been reported between 5-load and 2- or 3-load models [18,19,34]; however, there is some evidence that a greater number of loads may improve reliability of 1RM predictions [6,30]. This is essential when attempting to detect small changes in maximal strength. The specific loads used to develop a LVR may also impact on 1RM prediction validity and reliability; light loads are associated with higher movement velocities, which may inhibit movement coordination and impact on the validity of 1RM predictions, while heavier loads better represent an athlete’s maximal performance, but are more physically and mentally demanding. It is likely that heavy loads (≥80% or 1RM [19,31,41,42]) are more important to incorporate into the LVR than lighter loads to improve 1RM prediction validity [18], and that the loads used should cause velocities to differ by at least 0.5m·s^-1^. Whilst the inclusion of heavy loads to develop LVRs may cause a degree of fatigue [9,10], this can be somewhat overcome by only using single repetitions with heavier loads (≥70% 1RM). These single repetition sets with long rest periods (typically ≥2 minutes [6,18,19]) are unlikely to contribute significantly to fatigue, particularly in conditioned athletic cohorts. Multiple repetitions (typically three) are generally used for light loads (≤ 60% 1RM), with the fastest from each load included to develop the LVR [6,11,18]. Nevertheless, as some research has shown acceptable 1RM prediction validity and reliability for certain exercises such as the Smith machine bench press may be sufficient when loads >70% 1RM are not used [12], practitioners may also wish to consider the exercise being examined when determining the specific loads to be used.

Several studies included in this review proposed that 1RM predictions may be calculated from repetitions performed during warm-up to represent the maximal strength capabilities of an individual specific to that given day [6,9,15,17,30,34,35,38]. By prescribing the loads lifted during training based on this predicted 1RM value rather than a previously measured 1RM, it may be possible to prescribe a training stimulus more appropriate to the individual’s current performance capabilities [6,64]. While this approach seems intuitive, it is unknown whether other factors such as variations to sleep [65], caloric intake [66] and mental fatigue [67,68] may impact on 1RM estimates. As such, caution may be needed if using LVR-based 1RM predictions to quantify daily or session-specific maximal strength levels until these factors are investigated.

From the studies included in this systematic review, it seems that there are not substantial differences between exercises in the validity of predicting 1RM from the LVR. However, it possible that validity may be poorer in lower body exercises with greater technical demands (e.g., barbell back squats and deadlifts). It is also important to recognise that the validity and reliability of LVR-based 1RM predictions have only been assessed in a selection of exercises, and it is possible that these findings might not be replicated in alternative exercises. For example, research attention could be directed to dumbbell-based exercises that are commonly used in practice [2,3] but have not been used in LVR-based 1RM prediction research. Further, given only one study has examined these 1RM predictions in unilateral exercise, which was performed using a machine, and non-machine based unilateral exercises are common for hypertrophy and power training [69], research examining these exercises is warranted.

The studies included in this review indicate that either MCV, MPV or PCV can be used to develop valid and reliable LVR-based 1RM predictions. This is important for practitioners who may be using monitoring devices which do not provide data on all three of these metrics. When predicting 1RM for non-ballistic exercises such as those examined in this review, we recommend practitioners use MCV or MPV metrics measured with the most valid and reliable linear position transducer available. One caveat, however, is that the PCV has been suggested as most suitable for ballistic exercises [44]. Further research is required to clarify these findings, particularly considering that measured and predicted 1RM does not differ when calculated from MCV, whilst 1RM predictions made using the PVC are different to measured 1RM [44]. High-speed camera technology may also be used. Inertial measurement units may not be as valid or reliable for LVR-based 1RM predictions [19]. Practitioners should also provide instantaneous feedback regarding the velocity of all repetitions that are to be used to develop LVRs. Lastly, there is some evidence that individualised LVRs overestimate Smith machine bench press 1RM in relatively inexperienced lifters [40]. Further research is needed to confirm if this finding is consistent in trained lifters, or whether the findings extend to Smith machine or free-weight lower body exercise.

## Supporting information

S1 Checklist (DOCX)
